# Microbial and metabolic signatures of humanized microbiome mice associate with *Tlr2* expression and Th17 immune programming

**DOI:** 10.3389/fimmu.2026.1879544

**Published:** 2026-08-07

**Authors:** Alexis N. Cox-Holmes, George B. H. Green, Anna Claire E. Potier, Yong Wang, Dongquan Chen, Casey D. Morrow, Braden C. McFarland

**Affiliations:** 1 Department of Cell, Developmental and Integrative Biology, Heersink School of Medicine, University of Alabama at Birmingham (UAB), Birmingham, AL, United States; 2 Department of Genetics, Heersink School of Medicine, UAB, Birmingham AL, United States

**Keywords:** gut immunology, metabolomics, microbiome, single-cell RNA-sequencing, Th17

## Abstract

**Introduction:**

The gut microbiota plays a critical role in immune system development. However, whether normal variation in healthy human microbiota alone can establish distinct baseline immune states that influences responses to disease and therapy remains unclear.

**Methods:**

To address this, we employed a humanized microbiome (HuM) mouse model where fecal matter is transplanted from healthy human donors into gnotobiotic mice. Our previous studies found that HuM mouse lines have differential responses to glioma immunotherapy, so we sought to define how microbiota composition mediates host immunity prior to disease development.

**Results:**

Despite being genetically identical and disease-free, HuM mice exhibited distinct microbiota-driven immune profiles. Mice harboring responder-associated microbiota (HuM2) had higher proportions of gram-positive gut bacteria and decreased acetate and propionate compared to nonresponder-associated (HuM1) mice. Single-cell RNA-sequencing of colonic CD45^+^ cells from HuM2 mice had a higher portion of B cells and neutrophils, an increase in *Tlr2* in the lamina propria, and a systemic increase in Th17 cells compared to HuM1.

**Discussion:**

Together, these findings reveal a microbiota dependent program associating gram-positive enrichment and altered metabolite production to TLR2-associated immune activation and Th17 response. Our results demonstrate that human microbiome variation alone correlates with distinct baseline immune profiles that may predispose hosts to differential immunotherapy responses.

## 1 Introduction

The gut microbiota consists of all bacteria, virus, archaea, and fungi that inhabit the gastrointestinal tract. Bacteria make up the largest portion of the gut microbiota and are predominated by the phyla Bacteroidota and Firmicutes (1). Other phyla that compose the gut microbiota are Actinobacteria, Proteobacteria, Fusobacteria, Spirochaetota, and Verrucomicrobia (2). Numerous studies have revealed that gut microbiota are vital to the establishment, development, and modulation of the host’s immune system (3). Gnotobiotic, or microbiota-free, mice have immune irregularities including deficits in formation of gut associated lymphoid tissue, reduced IgA production, absence of mucosal associated invariant T cells, reduced Th17 development in the small intestines, expanded natural killer T cells, and more (3).

During infancy, the gut microbiota interacts with host immune cells in the gut using goblet-associated antigen passages (4). This allows the immune system to establish tolerance to beneficial microbes. Throughout life the intestinal immune system continues to sample the gut microbial antigens using specialize microfold cells and dendritic cells (5). Other microbial-immune interactions occur through pattern associated molecular patters from microbes and pattern recognition receptors on intestinal epithelial or immune cells. Classic examples of these are lipopolysaccharides (LPS) signaling through toll-like receptor 4 (TLR4) (6, 7), lipoteichoic acid (LTA) and peptidoglycans signaling through TLR2 (8–10), and bacterial flagellin signaling through TLR5 to induce an inflammatory response (11–13).

The gut microbiota also influences the immune system indirectly through microbial metabolites. Gut microbes break down dietary fiber to create short-chain fatty acids (SCFAs), such as acetate, propionate, and butyrate, which can activate immune cells or induce T regulatory cells (Tregs) (14–19). Bacteria also produce other metabolites such as medium and long chain fatty acids, tryptophan metabolites, and bile acids which are all known to modulate the immune system (20). Microbial byproducts and microbiota educated immune cells can circulate and influence the systemic immune response of the host.

Disturbances in the gut microbiota, known as gut dysbiosis, has been linked to inflammatory bowel disease, carcinogenesis, obesity, anxiety, and more (21, 22). Differences in gut microbiota have also been shown to alter response to cancer immunotherapies (23–26). A common challenge with modeling the role of the gut microbiota in diseases using lab mice is that there is only a 10% overlap in gut microbiota of humans and laboratory mice at the species level (27). To account for this, our lab utilizes a humanized microbiome (HuM) mouse model where fecal samples from healthy human donors are transplanted into gnotobiotic mice. These mice are then bred, to account for the underdeveloped immune system in gnotobiotic mice, and offspring used for disease modeling experiments (28). Furthermore, mice with different humanized microbiomes respond differently to antibiotics, corticosteroids, and immunotherapy for glioblastoma (24, 25, 29). The purpose of this study is to define how normal variation in healthy human gut microbiota composition mediates host immunity prior to disease development. To do this we sought to map differences in the microbiota, metabolome, and colonic and systemic immune phenotypes of HuM mice with microbiota derived from separate healthy human donors. For this study we have chosen to use two HuM lines that have previously responded differently to various glioma treatments, which are HuM1 (immunotherapy nonresponder) and HuM2 (immunotherapy responder) (24, 25, 29).

## 2 Materials and methods

### 2.1 Mice

Cryopreserved ceca from two lines of HuM mice, designated HuM1 and HuM2, from previous studies were utilized for transplantation into 10bitFoxP3.GFP.BL/6 gnotobiotic mice (24, 25, 29). Of the 5 HuM lines generated in Dees et al, only two lines (one immunotherapy responder and one nonresponder) were used for the reduction of variables (24, 25, 29). Each mouse received 100-200 μL of cecal matter in Cary-Blair media via oral gavage, resulting in the establishment of two distinct HuM lines, HuM1 and HuM2. These mice were then bred, and the offspring were used in all subsequent experiments. Experiments involving the mice were approved via the University of Alabama (UAB) Institutional Animal Care and Use Committee (#IACUC-22612 and #IACUC-22699) (24, 25, 29). No primary human stool samples were used in this study.

Though we have previously utilized and published this HuM model, these mice are new derivations from the previously described HuM1 and HuM2 (24, 25, 29). From alpha and beta diversity HuM1 samples (previously published and current) cluster together and separately from HuM2 samples. We present the new microbiome analysis of HuM1 and HuM2 to more accurately reflect the differences seen in the subsequent SCFA and immune cell characterization.

### 2.2 Fecal sample preparation and 16S rRNA sequencing

Total DNA was extracted from murine fecal samples using the Quick DNA Fecal/Soil Microbe Miniprep (ZYMO Research, CAT# D6010) according to the manufacturer’s instructions (25, 29). Isolated DNA was quantified and a purity assessment was performed on an Epoch microplate spectrophotometer (BioTek Instruments). An Illumina MiSeq (Illumina, Inc.) was used to preform high-throughput amplicon sequencing with the 250bp paired-end kits and by targeting the V4 hypervariable region of the bacterial 16S rRNA gene. Resulting sequences were demultiplexed and converted to FASTQ format. Raw sequence files were submitted to the National Center for Biotechnology Information (NCBI) Sequence Read Archive (SRA) under BioProject number: PRJNA1327931.

### 2.3 Taxonomic assignment and distribution

QIIME2 (2023.5) was used to determine the taxonomic profiles of fecal 16S data as previously described (25, 29). Briefly, fastq files were added using “qiime tools import” with the Cassava 1.8 paired-end demultiplexed format. Sequence file quality was checked via “qiime demux summarize”. DADA2 was used to denoise. Sequences for the features were determined by the q2-feature-table tabulate-seqs input. Amplicon sequence variants (ASVs) were aligned using the mafft program and a phylogenetic tree was generated using fasttree2. Taxonomic identifications were assigned to sequences using qiime feature-classifier and the “classify-sklearn” command to compare sequences against the silva-138-99-nb-classifier. After quality checking raw sequence files had 1,922,044 reads, and a total of 726 features were identified. Using phyloseq (v1.41.1) package in R, alpha diversity metrics were obtained by the estimate_richness() command. Beta diversity metrics were determined using the QIIME2 output. PCoA1 vs PCoA2 was calculated by the Bray-Curtis method and MicrobiotaProcess (v1.6.6) was used to add ellipses showing a confidence level of 0.9.

### 2.4 Predicted functional analysis

To predict potential functional profiles of the microbiota, Phylogenetic Investigation of Communities by Reconstruction of Unobserved States (PiCRUSt2, v2.5.2) was used and ggpicrust2 (v2.5.2) in R was used to determine significance and graph pathways. PiCRUSt2 uses ASVs from 16S data and maps them onto phylogenetic trees. It then uses a hidden-state prediction approach to estimate the functional gene profiles based on known gene repertoires of related organisms represented in the reference tree (30). The Kyoto Encyclopedia of Genes and Genomes (KEGG) functional profiles were obtained utilizing ggpicrust2. Predicted gees are adjusted based on copy number and relative abundance of the ASV and then used to reconstruct the expected metagenomics composition of microbial communities (30).

### 2.5 Shotgun metagenomic analysis

To complement the 16S sequencing, shotgun metagenomics sequencing was performed on one pooled (n=4/line) fecal DNA sample from each line. One pooled sample was used due to limited resources. Sequencing was performed by the UAB microbiome sequencing core. Shotgun metagenomic sequencing reads were analyzed using MetaPhlAn to determine microbial taxonomic profiles (31). Raw FASTQ files underwent quality control and removal of host-derived sequences by alignment to the mouse reference genome. Non-host, high-quality reads were processed by MetaPhlAn using a clade-specific marker gene database to classify microbial taxa. Resulting tables of taxonomic abundance were then used for downstream analyses of relative abundance comparisons. Data visualization was performed using the following Python packages: *pandas* for data manipulation and *seaborn* and *matplotlib* for visualization and plotting.

### 2.6 Cecal sample preparation and metabolite analysis

Whole ceca were harvested from HuM mice and cryopreserved. Samples were sent to the Metabolomics and Proteomics Laboratory at UAB where targeted liquid chromatography tandem mass spectrometry (LC-MS) analysis was performed on short and medium chain fatty acids: acetic acid, butyric acid, isobutyric acid, propionic acid, valeric acid, isovaleric acid, hexanoic acid, and n-heptanoic acid. Briefly, ceca were ground into a powder in liquid nitrogen and metabolites were extracted with methanol. LC-MS was performed on a Shimadzu Prominence HPLC system and SCIEX 4000 mass spectrometer using a Kinetix C18 column.

### 2.7 Isolation, FACS sorting, and single cell RNA sequencing of colon lamina propria lymphocytes

Colons were harvested and mucus and epithelium was removed by shaking samples in 2% fetal bovine serum (FBS), 1mM dithiothreitol (Sigma, CAT# D9779-5G), and 5mM ethylenediaminetetraacetic acid in Hank’s Balanced Salt Solution (HBSS) at 37˚C for 15 min twice. Colon tissue was minced and digested in collagenase/DNAse solution (3mg/mL Collagenase D, Roche REF# 11088858001: 50μg/mL DNAse, Worthington CAT# L6002139) in R10 medium (RPMI 1640 media, 10% FBS, 2mM L-glutamine, 50U/mL Penicillin/Streptomycin, 0.1% 2 β-mercaptoethanol, 1mM Na Pyruvate, 1x Nonessential Amino Acids, and 10mM HEPES). Samples were filtered through a 100μm filter and mononuclear cells were separated over a 75%/40% discontinuous Percoll gradient at 700g for 20 min, no brake. Cells were collected from the buffy layer and washed in R10 before staining. Single cell suspensions were incubated with Fc block (Bio X Cell, CAT# BE0307, 2.4G2) for 15 min at room temperature. Next cells were incubated with Biolegend TotalSeq hashtag antibodies (“ACCCACCAGTAAGAC”,”GGTCGAGAGCATTCA”, “CTTGCCGCATGTCAT”, and “AAAGCATTCTTCACG”) (CAT# 155831, 155833, 155835, 155837), CD45-APC (Biolegend, CAT# 103112; 1:200), and Zombie Aqua fixable viability die (Biolegend, CAT# 423101; 1:500) for 30 min at 4˚C. Cells were sorted for live CD45^+^ single cells on a BD FACS ARIA SORP in the UAB Flow Cytometry and Single Cell Core Facility into cold PBS with 0.04% BSA. Three to four samples per group were pooled to increase cell counts. This was necessary as it is recommended to have greater than 3000 cells per sample for single cell RNA-sequencing. Naïve colons have fewer immune cells than disease models, thus even after pooling multiple colons some of these samples were still less than 3000 cells (estimated cell counts in **Supplementary Figure 3**). Samples were sent to the UAB Single Cell Core Facility where it was prepped using 10x protocol and run on a NovaSeq 5000 System from Illumina. Raw sequence files were submitted to the NCBI under BioProject number: PRJNA1354473.

### 2.8 Single cell analysis

Demultiplexing, alignment, and filtering were performed through cell ranger (v7.10). Cells expressing <200 genes and >5% mitochondrial DNA were removed. A total of 15,954 cells from 8 samples were analyzed. Raw data was imported into R and log transformed using Seurat v5 package “NormalizeData” function. Seurat was also used for cell clustering and dimension reduction. “findallmarkers” was used to determine differentially expressed genes (DEGs) for the 17 clusters and cell annotations were assigned manually from the top DEGs. Cell proportions were calculated as the percentage of the cell type relative to total cells. To compare DEGs within a particular cell type, the Seurat FindMarkers() function was used and the genes were graphed using RidgePlot() in Seurat or sc.pl.dotplot() in Scanpy. For pathway analysis, Single Cell Pathway Analysis (SCPA) v1.6.1 was used. To compare predicted cell-cell signaling, CellChat was used.

### 2.9 Flow cytometry phenotyping

For immune phenotyping experiments, lymph nodes (LN) were processed through a 100μm filter. Cells were incubated with Fc block (Bio X Cell, CAT# BE0307, 2.4G2) for 10 min, then incubated with Zombie Aqua fixable viability dye (Biolegend, CAT# 423101; 1:500) and surface antibodies [CD8a-PerCP-Cy5.5 (Biolegend, CAT# 100734; 1:200), CD45-APC-Cy7 (Biolegend, CAT# 103115; 1:200), CD3-Bv605 (Biolegend, CAT# 100237; 1:100), CD4-Bv650 (BD Horizon, CAT# 563232; 1:200)] for 30 min at 4˚C. Cells were then fixed and permeabilized with the eBioscience™ Foxp3/Transcription Factor Fixation/Permeabilization kit (CAT# 00-5521-00) before incubation with intracellular staining for transcription factors and cytokines [FoxP3-FITC (eBioscience, CAT# 11-5773-82; 1:100), RORγt-APC (eBioscience, CAT# 17-6981-80; 1:50), Granzyme B-Pacific Blue (Biolegend, CAT# 515407; 1:50), IFNγ-PE-Cy7 (Biolegend, CAT# 505826; 1:100), GMCSF-PE (eBioscience, CAT# 12-7331-82; 1:100)] for 30 min at room temperature. Cells were phenotyped on the Attune NxT at the UAB Flow Cytometry and Single Cell Core Facility and analyzed using FlowJo10 software. Th17 cells were gated as: cells (FSC-A vs SSC-A), single cells (FSC-H vs FSC-A), live (Zombie Aqua negative), CD45+, CD3+, CD4+, FoxP3-, then Rorγt+.

### 2.10 Naïve CD4 T cell polarization assay

Naïve CD4^+^ T cells were enriched from spleens using the Mouse Naïve CD4^+^ T Cell Kit from Stem Cell Technologies (CAT# 19765A). Naïve CD4+ T cell polarization assays were performed as previously described (32). Briefly, cells were cultured at a density of 10^6^ cells/mL in R10 then activated with plate-bound anti-CD3 (10 μg/mL: Biolegend, CAT #100238) and anti-CD28 (1 μg/mL; Biolegend, CAT# 102116). Th17 polarizing conditions were IL-6 (20 ng/mL; Biolegend, CAT# 575702), IL-23 (10 ng/mL; Biolegend, CAT# 589002), TGFB1 (2.5 ng/mL; Biolegend, CAT# 763102), anti-IL-4 (10 μg/mL; Biolegend, CAT# 504102), anti-IFNγ (10 μg/mL; Biolegend, CAT# 505802). After 72 hours, cells were stimulated with PMA (Sigma, CAT# P8139-1MG) and Ionomycin (Sigma, CAT# I0634-1MG) for 4 hours in the presence of BD GolgiStop (CAT# 51-2092K2). Cells were incubated with Fc block and then with Zombie Aqua fixable viability dye (1:500) and CD4-Pacific Blue (1:200; Biolegend, CAT# 100428) for 30 min at 4˚C. Cells were fixed and permeabilized using the BD Cytofix/Cytoperm™ Fixation/Permeabilization Kit (CAT# 554714) before being incubated with intracellular antibodies [IFNγ-PE-Cy7 (Biolegend, CAT# 505826; 1:100), IL17a-APC (Biolegend, CAT# 506916; 1:100), FoxP3-FITC (eBioscience, CAT# 11-5773-82; 1:100)]. Flow cytometry was ran and analyzed as stated in previous subsection. Cells were gated as: cells, single cells (FSC-H vs FSC-A), live (zombie aqua negative), CD4+, IL-17+.

### 2.11 Statistics

For microbiome analysis, statistical significance for alpha diversity indices were determined using Wilcoxon rank sum test. Statistics for beta diversity were determined using Permutational Multivariate Analysis of Variance (PERMANOVA) and Permutational Multivariate Analysis of Dispersion (PERMDISP). For determining significance of DEGs in single cell RNA-sequencing, Seurat FindMarkers() with DESeq2 test was used. For all other analyses, either student’s t-test or Wilcoxon rank sum test (if normal distribution failed) was used to determine significance. For all statistical analyses, alpha was set to 0.05. Graphs were made in R or GraphPad prism.

## 3 Results

### 3.1 HuM1 and HuM2 mice have significantly different gut microbial compositions

**Figure 1:**
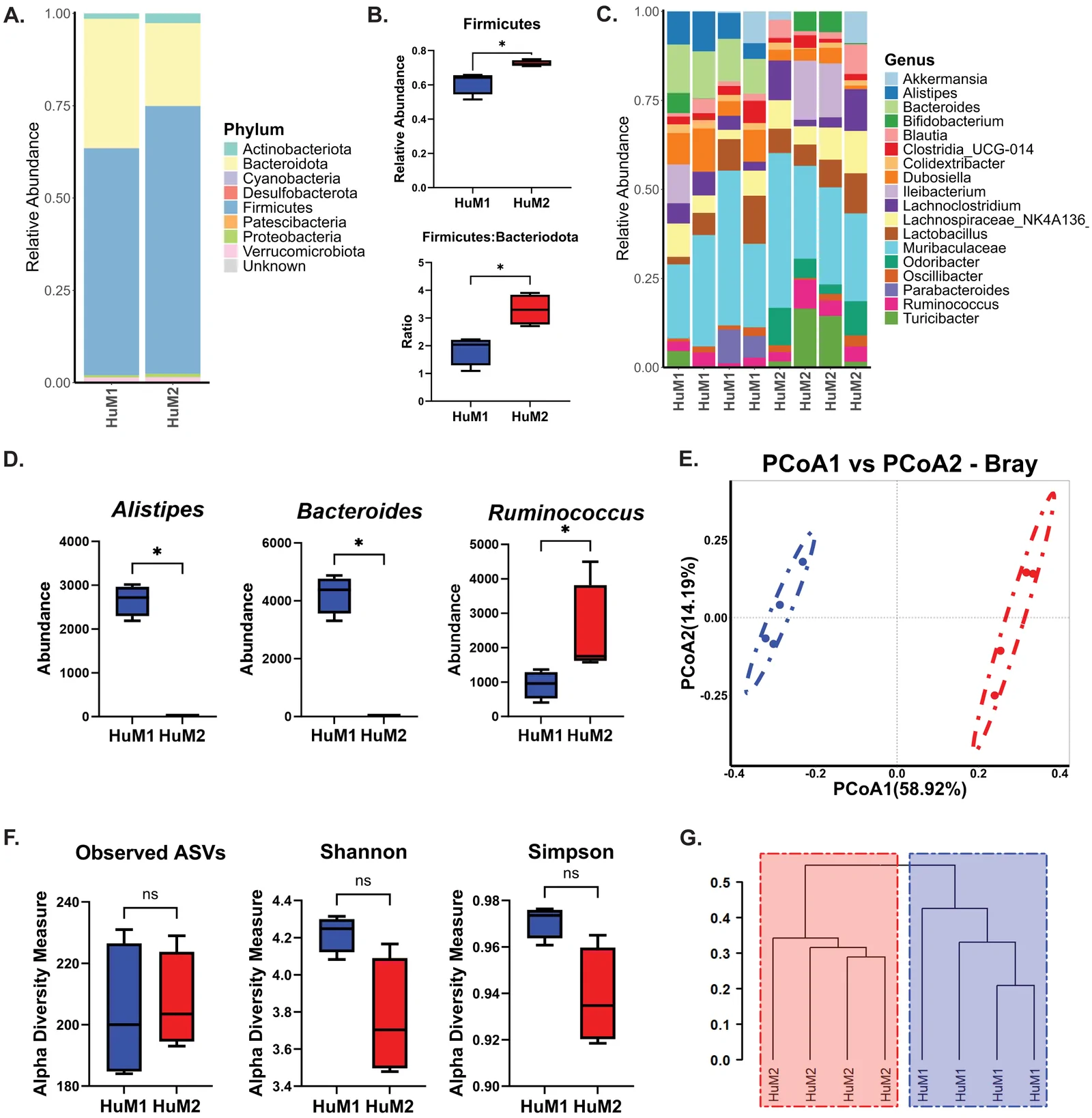
HuM1 and HuM2 had significantly different gut microbiomes. **(A)** The relative abundance of the bacteria Phyla across HuM1 and HuM2 fecal samples. **(B)** The relative abundance of Firmicutes phyla (top) and Firmicutes: Bacteroidota (F/B) ratio (bottom) differences between HuM1 and HuM2 samples. **(C)** The relative abundance of the top 20 taxa at the Genus level for HuM1 and HuM2 samples. **(D)** Boxplots showing pairwise analysis of significantly different genera between HuM1 and HuM2 samples. **(E)** PCOA plot illustrating beta diversity using Bray-Curtis metrics. **(F)** Alpha diversity measurements (Observed ASVs, Shannon diversity, and Simpson diversity) compared between HuM1 and HuM2 samples. **(G)** Dendrogram illustrating unique microbial compositions between the fecal samples of HuM1 and HuM2 mice. All pairwise comparisons were analyzed with Wilcoxon rank sum test (* p<0.05). Each point represents a pooled sample of n=3–4 for a total of n=15 mice (8 male, 7 female) per line.

Fecal samples were collected from naïve HuM1 and HuM2 mice and 16S sequencing performed. At the phylum level, both HuM1 and HuM2 were predominated by Firmicutes, Bacteroidota, and Actinobacteria (**Figure 1A**), and HuM2 had a higher abundance of Firmicutes and a higher Firmicutes to Bacteroidota (F/B) ratio (**Figure 1B**). The top 20 genera (filtered to remove “Unknown” and “Other”) were graphed in **Figure 1C**. HuM1 had significantly higher abundance of the gram-negative genera *Alistipes* and *Bacteroides*, and HuM2 had a significantly higher abundance of the gram-positive genera *Ruminococcus* (**Figure 1D**). Bray-Curtis analysis of HuM1 and HuM2 revealed significantly different clustering of the microbiomes (**Figure 1E**). PERMANOVA statistics supported this dissimilarity between groups (R2 = 0.59, p = 0.0297), and PERMDISP revealed no significant dispersion (p>0.05). Significant differences in richness between HuM1 and HuM2 were not observed (**Figure 1F**). Lastly, a dendrogram further revealed distinct separation of the microbiome samples between the two lines (**Figure 1G**).

For a descriptive observation of the species composition of the two microbiomes, shotgun sequencing was performed on the HuM1 and HuM2 mice (one pooled sample of n=4 each). The top 10 species for each pooled sample are shown in **Supplementary Figure 1** and full list of species can be found in **Supplementary Table 1**. The HuM1 sample had high abundance of unclassified *Bacteroidales bacterium* (t_SGB27761 and t_SGB4185) and *Bacteroides acidifaciens* with *Dubosiella newyorkensis*, and *Paramuribaculum intestinale* also being in the top 10 most abundant species (**Supplementary Figure 1**). The HuM2 sample had high abundance of unclassified *Lachnospiraceae* (GGB27876_SGB40310), *Erysipelotrichaceae_bacterium_NYU_BL_F16*, and unclassified *Bacteroidales* (GGB27849_SGB40283) with *Bifidobacterium pseudolongum*, *Ligilactobacillus murinus*, and *Turicibacter* sp *1E2* also being in the top 10 most abundant species (**Supplementary Figure 1**). Overall, these data support that HuM1 and HuM2 have significantly different gut microbiomes.

### 3.2 Predicted functional analysis of HuM1 and HuM2 microbiota revealed significant metabolic pathway differences

**Figure 2:**
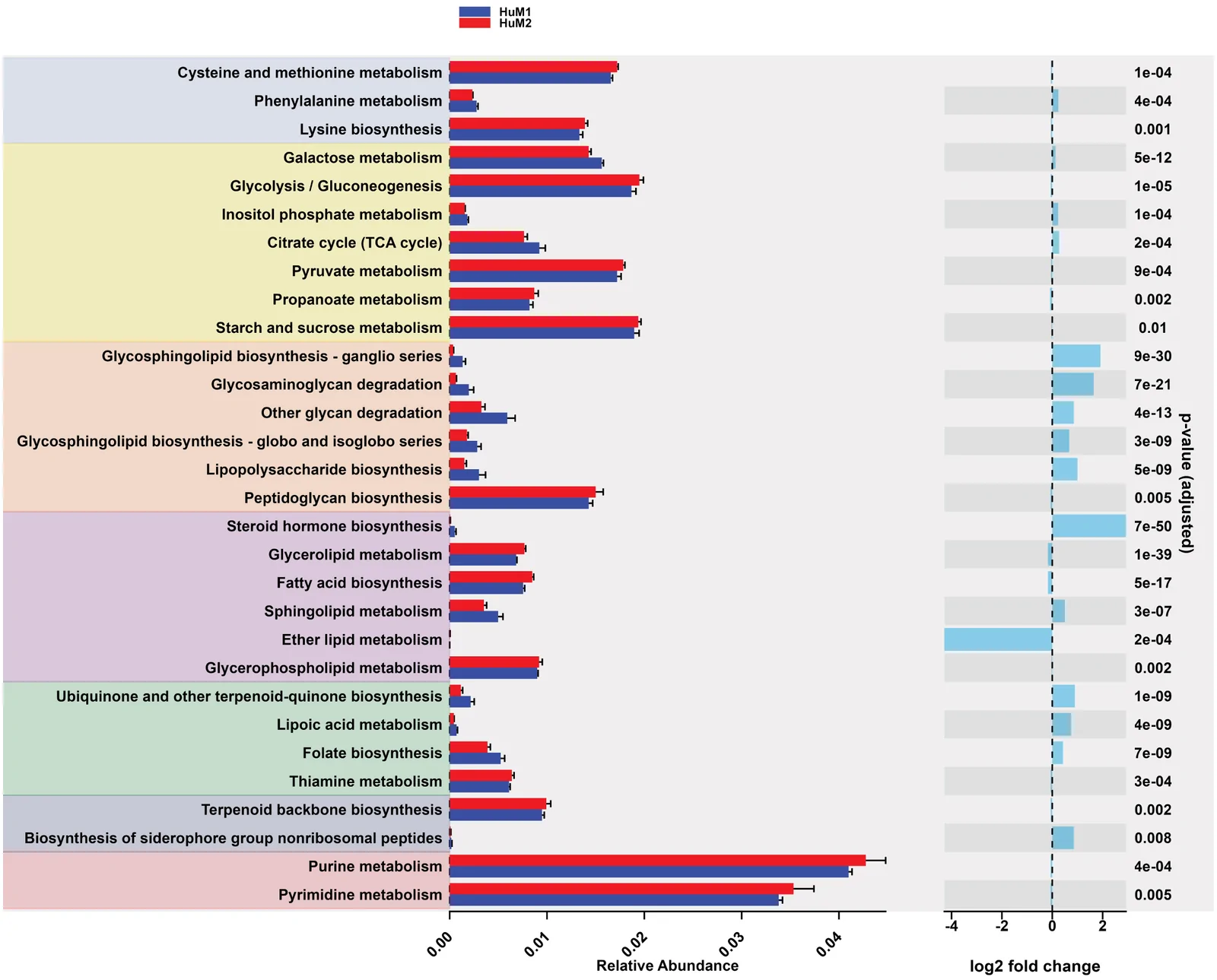
HuM1 and HuM2 had differences in predicted metabolic pathways. Predicted functional pathways were determined on the 16S rRNA sequencing data from the HuM1 (n=15; 8 male, 7 female) and HuM2 (n=15: 8 male, 7 female) fecal samples using PiCrust2 and displayed are the relative abundances, log2 fold changes, and p-values of the significantly different metabolic pathways. Metabolic pathways are color coded by sub-category: Amino acid metabolism (grey), Carbohydrate metabolism (yellow), Glycan biosynthesis and metabolism (orange), Lipid metabolism (purple), Metabolism of cofactors and vitamins (green), Metabolism of terpenoids and polyketides (blue), Nucleotide metabolism (red).

To assess the potential functional microbial pathway differences between the HuM mice, PiCrust2 analysis was performed on the 16S data. The analysis revealed many significant differences in predicted KEGG pathways between the groups. Notably, HuM2 microbiota were enriched in pathways associated with peptidoglycan biosynthesis and production of lipoteichoic acid (LTA) precursors (glycerolipid metabolism and terpenoid backbone biosynthesis), consistent with the greater abundance of gram-positive taxa in these mice (**Figure 2**). Conversely, HuM1 microbiota were enriched in LPS biosynthesis, consistent with their greater abundance of gram-negative taxa. (**Figure 2**). The HuM2 microbiota had significantly higher predicted starch and glycolysis, pyruvate metabolism, sucrose metabolism, and purine/pyrimidine metabolism (**Figure 2**). Alternatively, the microbiota of HuM1 mice had higher predicted TCA cycle, glycan degradation, steroid hormone biosynthesis, sphingolipid metabolism, and folate biosynthesis (**Figure 2**). Overall, this confirms that the different gut microbiomes of HuM1 and HuM2 mice have predicted alterations in numerous metabolic pathways, which may impact the microbe-immune interactions and the immune development of the colons of these mice.

### 3.3 HuM mice had significant differences in cecal short- and medium-chain fatty acid metabolites

**Figure 3:**
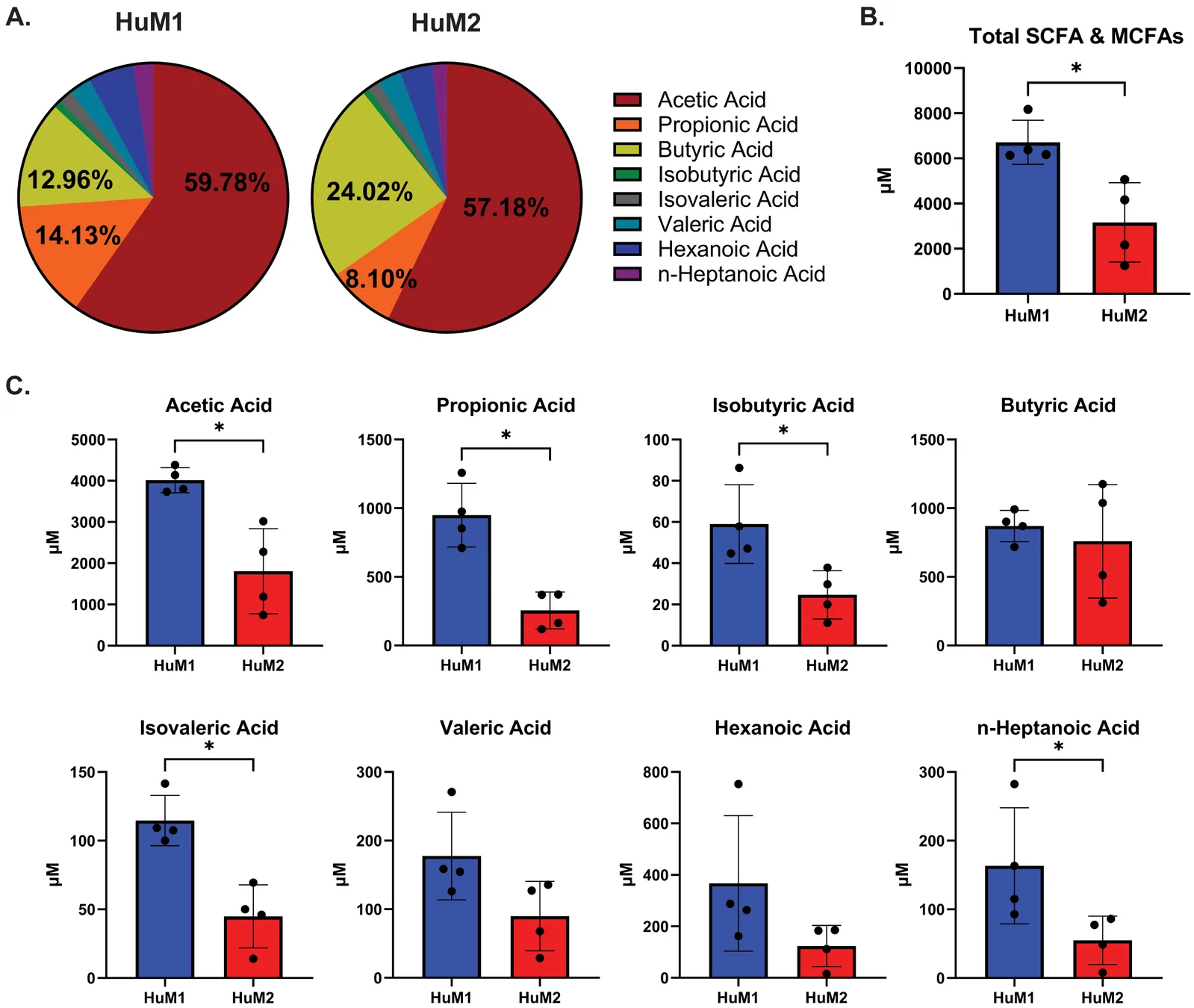
HuM mice had significant differences in cecal short- and medium-chain fatty acid (SCFA and MCFA) metabolites. Targeted metabolomics was performed on the cecal matter of HuM2 and HuM1 mice. **(A)** Pie chart breakdown on the relative proportions of the SCFAs/MCFAs in the HuM1 and HuM2 cecal matter. **(B)** Bar plot of the concentration of total SCFA and MCFAs measured. **(C)** Bar plots of the concentrations of each individual metabolite measured between HuM1 and HuM2 samples. All pairwise comparisons were analyzed with Wilcoxon rank sum test (* p<0.05). Each point represents a pooled sample of n=3–4 for a total of n=15 mice (8 male, 7 female) per line.

Targeted metabolomics using LC-MS was performed on cecal matter from HuM1 and HuM2 mice, to determine differences in microbiota derived SCFAs (acetic acid, butyric acid, isobutyric acid, propionic acid, valeric acid, isovaleric acid) and medium chain fatty acids (MCFAs; hexanoic acid and n-heptanoic acid). The three most abundant SCFAs were acetic acid, propionic acid, and butyric acid (**Figure 3A**). These SCFAs are thought to be found in a 60:20:20 ratio in healthy murine and human guts. Chi Squared analysis of ratios of these metabolites revealed that HuM2 differed significantly from the expected ratio (x2 = 8.426, df = 2, p = 0.015) (**Supplementary Table 2**). HuM2 had a lower concentration in total SCFA and MCFAs from the panel (**Figure 3B**). Specifically, HuM2 had significantly lower concentrations of acetic acid, propionic acid, isobutyric acid, isovaleric acid, and n-heptanoic acid *(***Figure 3C**). Therefore, HuM1 and HuM2 have significantly different cecal metabolite compositions.

### 3.4 Single-cell RNA sequencing analysis of immune cells in the naïve colon reveal distinct cell populations

**Figure 4:**
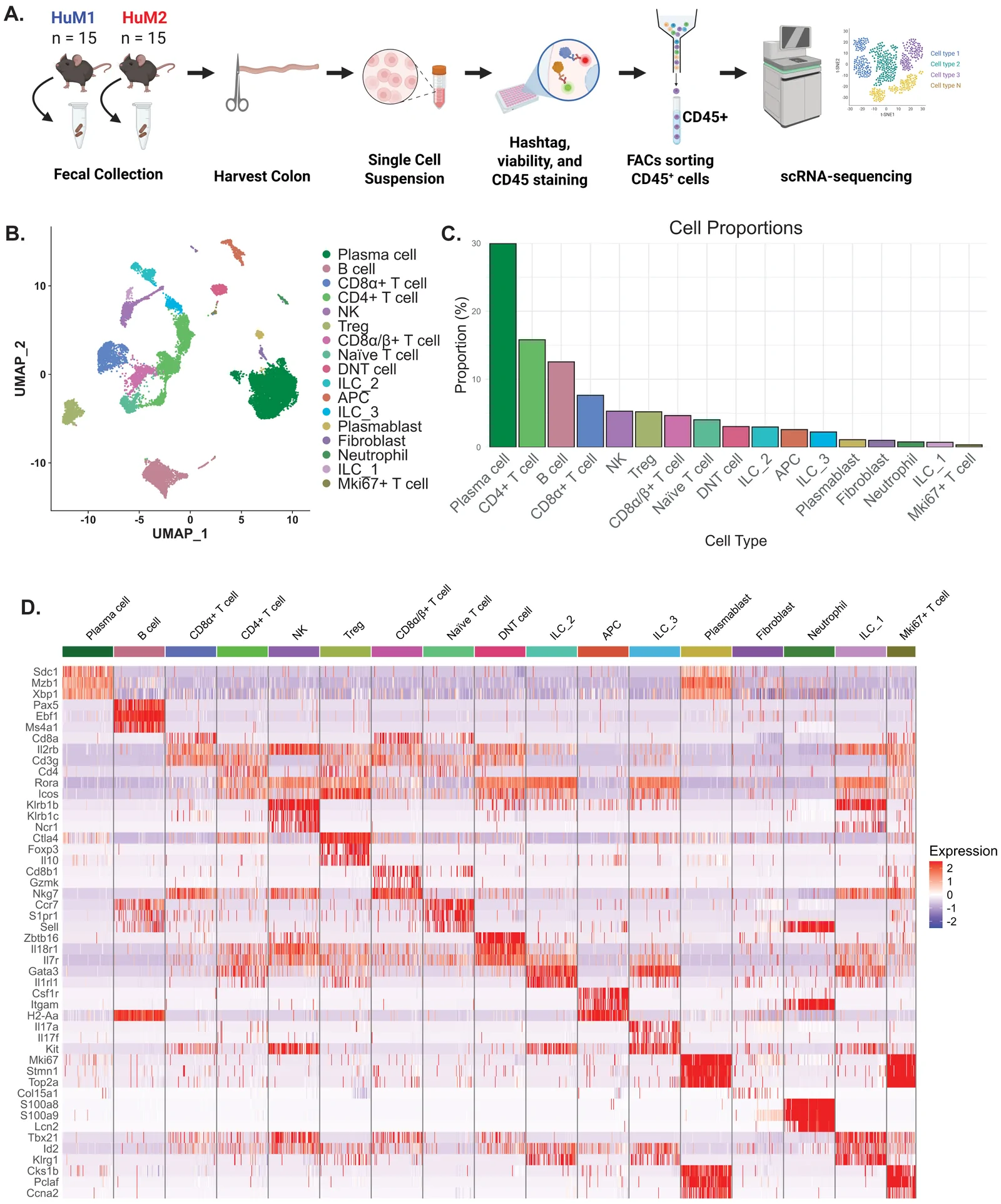
Single-cell RNA sequencing analysis of immune cells in the naïve colon reveal distinct cell populations. **(A)** Schematic of single-cell experiment. **(B)** Combined UMAP of all HuM1 and HuM2 samples generated by Seurat (v5.1.0). **(C)** Bar plot of cell proportions across all samples combined. **(D)** Heatmap generated by Seurat (v5.1.0) of 3 upregulated genes used for cell type identification of each cluster. **(A)** Created in BioRender. Cox-Holmes, A (2026). https://BioRender.com/ernsan5.

In order to determine if there were baseline differences in the colonic lamina propria immune environment of our two lines of HuM mice, we performed scRNA-seq on the CD45^+^ colon lamina propria cells of HuM1 and HuM2 mice (**Figures 4A**; **Supplementary Figure 2A, B**). We analyzed a total of 15,954 cells, and 2,000 features, and visualization of all cells were performed using uniform manifold approximation and projection (UMAP). 17 clusters were identified: Plasma cell (*Sdc1^+^, Mzb1^+^*), B cell (*Pax5^+^, Ms4a1^+^)*, CD8α^+^ T cell (*Cd3g^+^, Cd8a^+^, Cd8b^-^*), CD4^+^ T cell (*Cd3g^+^, Cd4^+^*), Natural killer cells (NK; *Klrb1b^+^, Klrb1c^+^*), Treg (*Cd4^+^, FoxP3^+^*), CD8α/β^+^ T cell (*Cd3g^+^, Cd8a^+^, CD8b^+^*), Naïve T cell (*Cd3g^+^, Ccr7^+^, S1pr1^+^*), Double negative T cell (DNT; *Cd3g^+^, Cd4^-^, Cd8^-^*), Innate lymphoid cell cluster 2 (ILC_2; *Il7r^+^, Gata3^+^*), Antigen presenting cell (APC; *Csf1r1^+^, Itgam^+^*), ILC cluster 3 (ILC_3; *Il17a^+^, Il17f^+^*), Plasmablast (*Sdc1^+^, Mki67^+^*), Fibroblast (*Col15a1^+^*), Neutrophil (*S100a8^+^, S100a9^+^*), ILC cluster 1 (ILC_1; *Tbx21^+^, Id2^+^*), and Mki67^+^ T cell (*Cd3g^+^, Mki67^+^*) (**Figures 4B–D**). **Supplementary Table 3** contains a complete list of genes used to identify clusters.

### 3.5 Significant differences in B cells, ILCs, T cells, and neutrophils between HuM1 and HuM2 colons

**Figure 5:**
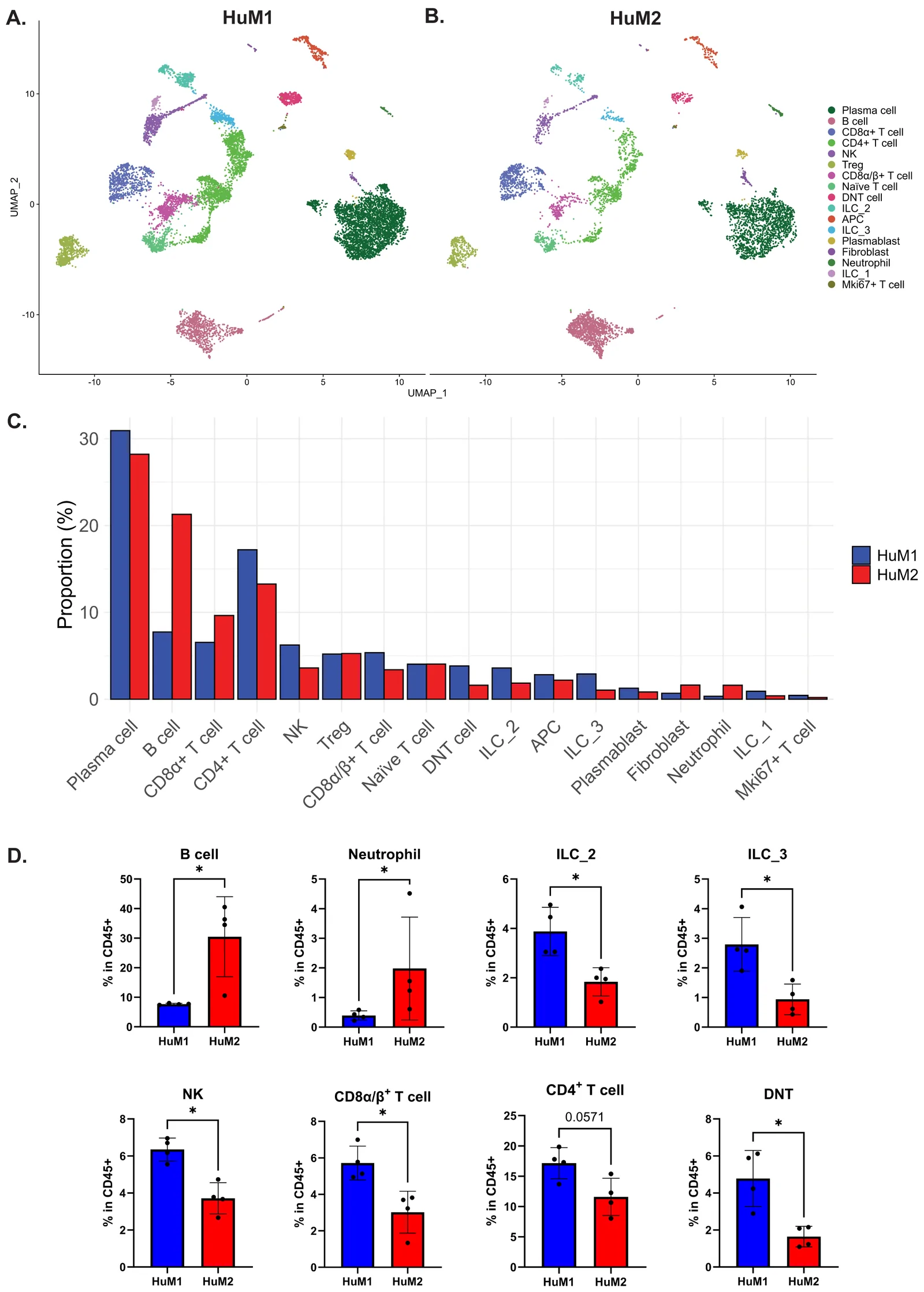
There were significant differences in B cells, ILCs, T cells, and neutrophils between HuM1 and HuM2 colons. Seurat UMAP of immune cell clusters in **(A)** HuM1 and **(B)** HuM2 colons. **(C)** Bar plot showing the differences in proportions of colonic immune cell types between HuM1 and HuM2 samples. **(D)** Bar plots showing pairwise comparisons of significantly different, or trending, immune cell proportions between HuM1 and HuM2 samples. All pairwise comparisons were analyzed with Wilcoxon rank sum test (* p<0.05). Each point represents a pooled sample of 3–4 mice, for a total of n=15 mice (8 male, 7 female) per line.

Next, we examine the differences between the immune landscape of the colon lamina propria of HuM1 and HuM2 mice. By both scRNA-seq and flow cytometry, the colons of HuM2 mice had a higher frequency of adaptive immune cells and lower frequency of innate immune cells compared to HuM1 colons (**Supplementary Figures 2C, D**). **Figures 5A, B** shows the UMAP of the colonic immune cells separated by HuM1 (**Figure 5A**) and HuM2 (**Figure 5B**). Colonic immune cells in these mice were predominated by Plasma cells and B cells, and differences in frequencies of the immune subsets by line can be observed (**Figure 5C**). Pairwise statistics were preformed between HuM1 and HuM2 colonic immune subsets and those of significance or trending were graphed (**Figure 5D**). HuM2 mice had significantly higher frequencies of colonic B cells and neutrophils, but significantly lower frequencies of colonic ILC_2, ILC_3, NK, CD8α/β^+^ T cells, and DNTs (**Figure 5D**). This data suggests that colonization with two different healthy human microbiota in HuM1 and HuM2 mice can lead to significantly different baseline colonic immune landscapes.

### 3.6 HuM2 mice had higher expression of *Tlr2* in the colon lamina propria immune cells

**Figure 6:**
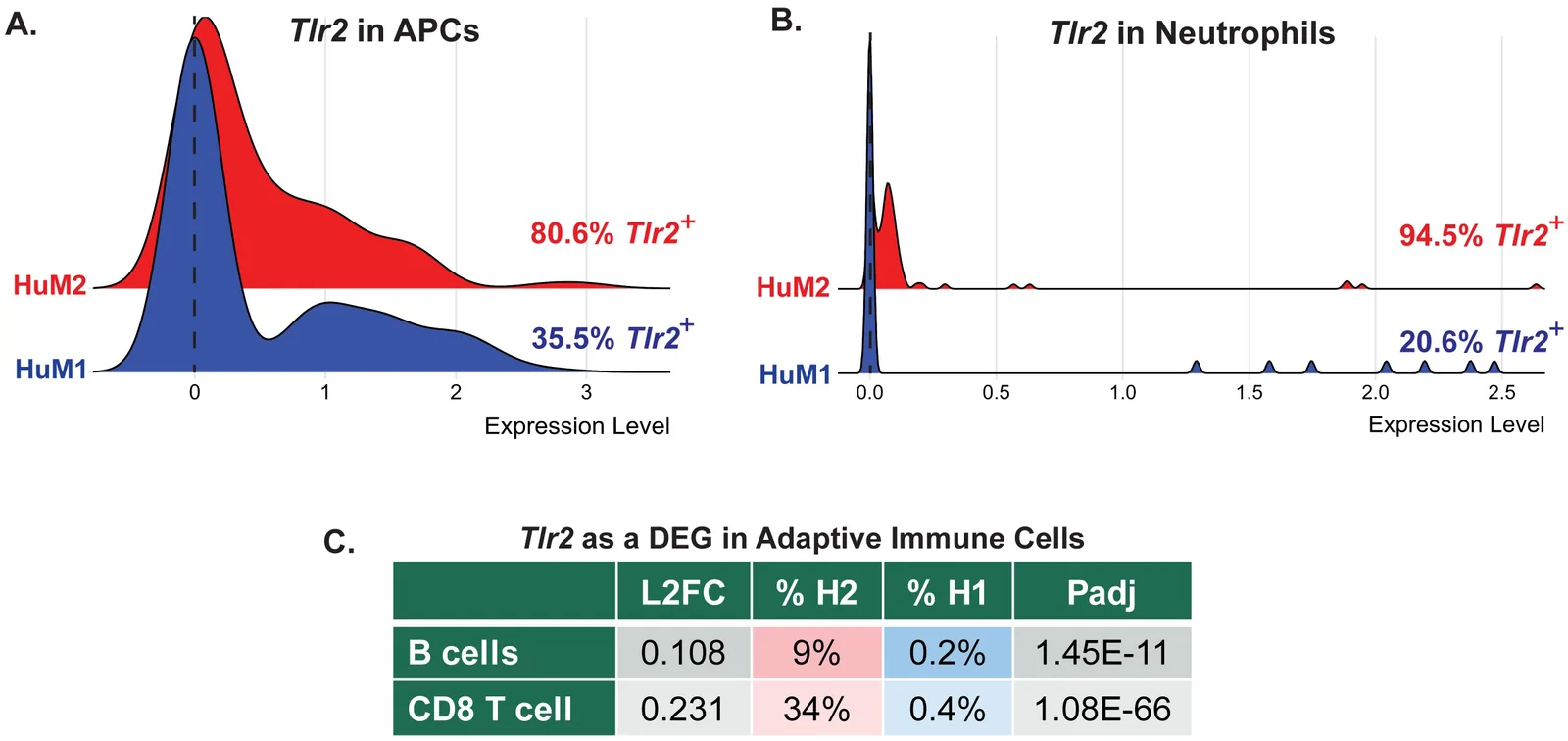
*Tlr2* mRNA differences between HuM1 and HuM2 samples. Ridge plots of *Tlr2* expression in **(A)** APCs and **(B)** Neutrophils. **(C)** Log2 fold change (L2FC), frequency of cells expressing Tlr2 and adjusted p-value (Padj) for *Tlr2* in B cells and CD8α/β^+^ T cells of HuM1 and HuM2 mice.

Because HuM2 mice had higher levels of gram-positive microbes, as well as in increase in predicted peptidoglycan biosynthesis (**Figure 2**) and predicted pathways that produce LTA (glycerolipid metabolism and terpenoid backbone biosynthesis) (**Figure 2**), we next wanted to see if these predicted pathway differences resulted in measurable differences in *Tlr2* expression in the immune cells in the colon. There was higher expression level and frequency of expression of *Tlr2* in colonic immune cells of HuM2 mice (**Figures 6A, B**), and *Tlr2* was a DEG in APCs, Neutrophils, B cells and CD8 T cells in HuM2 mice (**Figures 6A–C**). While *Tlr2* is primarily expressed in innate cells, it has been observed on B cells and T cells during activation and inflammatory conditions (33–37). Interestingly, 30% of CD8 T cells in the colons of HuM2 mice expressed *Tlr2* compared to 0.4% of HuM1 colonic CD8 T cells *(***Figure 6C***).* Overall, this data indicates that HuM2 mice have an increase in *Tlr2* expression in their colons, specifically in the immune cells, consistent with the increase in gram-positive gut microbiota.

### 3.7 HuM2 mice had a systemic increase in Th17 expression

**Figure 7:**
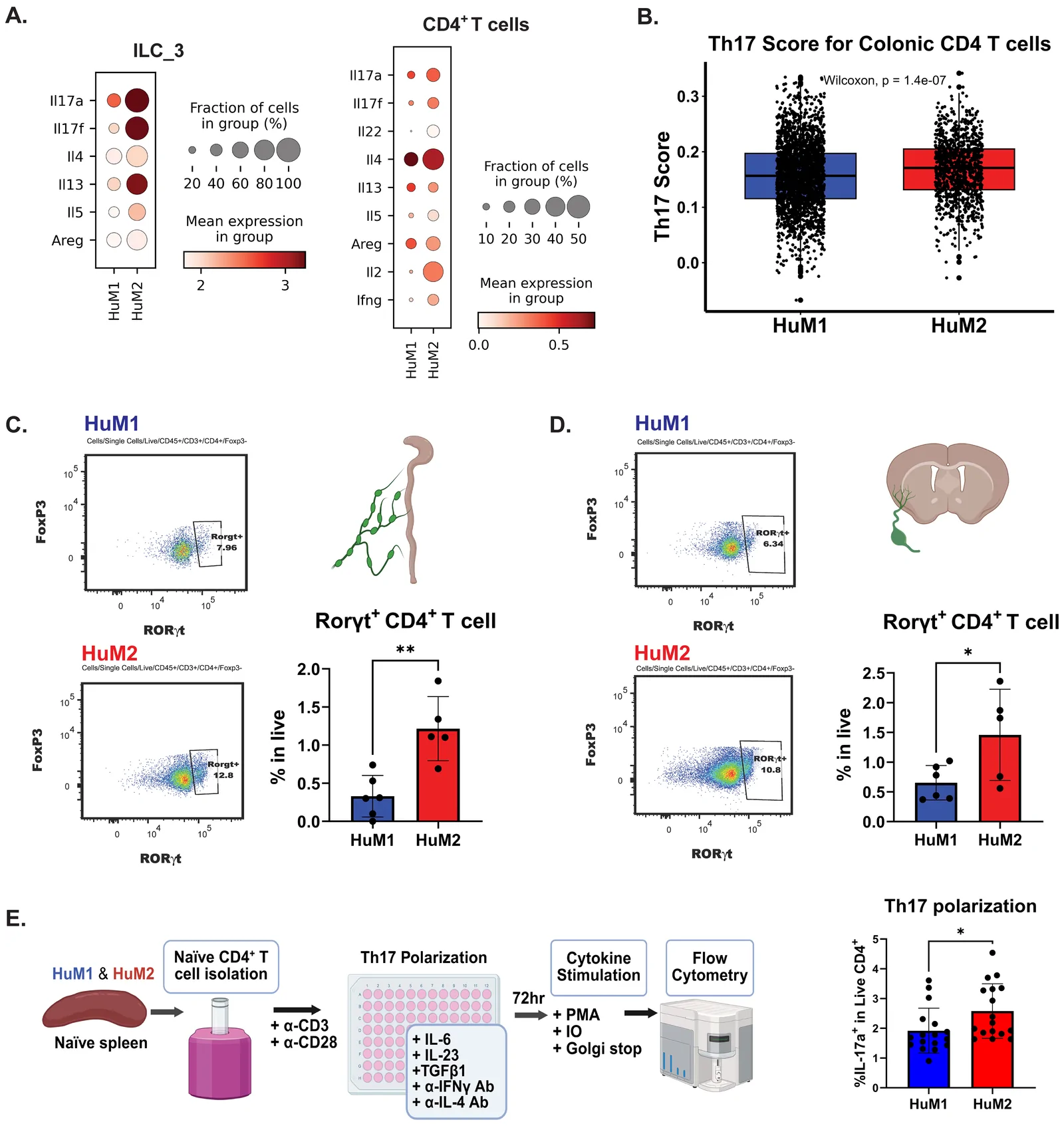
Systemic differences in Th17 phenotype between HuM1 and HuM2 mice. **(A)** Scanpy (v1.10.2) dotplots of cytokine expression within CD4^+^ T cells (right) and ILC_3 cells (left) between HuM1 and HuM2 samples. **(B)** Th17 phenotype scores of colonic CD4^+^ T cells between HuM1 and HuM2 mice. Th17 score assigned using Seurat (v5.1.0) AddModuleScore() and analyzed with Wilcoxon rank sum test. Single cell analysis came from cells of n=15 mice (8 male, 7 female) per line. **(C, D)** Flow cytometry analysis and bar plots of the frequencies of Rorγt+ CD4^+^ T cells in the **(C)** gut draining LNs (mesenteric, iliac, and caudal LNs) and **(D)** cervical LNs of HuM1 (n=6; 2 female, 4 male) and HuM2 (n=5; 2 female, 3 male) mice. Pairwise comparisons were analyzed with Wilcoxon rank sum test (* p<0.05, ** p<0.01). **(E)** Th17 polarization assay of naïve splenic CD4+ T cells from HuM1 (n=17, 11 male, 6 female) and HuM2 (n=17, 10 male, 7 female) mice across 4 experiments. Bar plot shows %IL-17^+^CD4^+^ T cells in live cells (t-test, *p<0.05). **(C–E)** Created in BioRender. Cox-Holmes, **(A)** (2026) https://BioRender.com/ernsan5.

Th17 cells are predominantly located in the intestinal lamina propria and play an important role in the gut microbiota-immune homeostasis of the gut. For this reason, we wanted to examine the Th17 phenotypes in these HuM mice. We found that ILC3s had the highest *Il17* expression of all cell subsets. Within ILC3s, HuM2 mice had much higher mean expression of *Il17a/f* and percentage of cells expressing these genes compared to HuM1 (**Figure 7A**). Within the colon CD4^+^ T cells, a higher fraction of these cells in HuM2 expressed Th17 cytokines *Il17a* and *Il17f* (**Figure 7A**). Alternatively, Th2 cytokine genes *Il4, Il13*, and *Areg* were expressed higher in HuM1 CD4^+^ T cells (**Figure 7A**). While IL-17 expression was lower in CD4 T cells compared to their innate counterpart, we used a list of Th17 genes (including *Il17a, Il17f, Ccl20, Dhrs9, Hif1a, Il22, Il23r, Rora*, and more*)* from Hu et al. to create a Th17 phenotype score for the CD4 T cell populations (38). HuM2 mice had a significantly higher Th17 score for the colonic CD4 T cells (Wilcoxon p<0.05) (**Figure 7B**). To validate these changes in Th17 phenotype, we isolated cells from the LNs of new sets of naïve HuM1 and HuM2 mice. We then examined the frequency of Th17 cells in the gut draining LNs (mesenteric, iliac, and caudal) and the cervical LNs as a distal site. At both sites HuM2 mice had a higher abundance of Th17 cells (Rorγt^+^CD4^+^ T cells) (**Figures 7C, D**). Using a naïve CD4^+^ T cell polarization assay, splenocytes from naive HuM2 mice had a higher frequency of Th17 polarization compared to those from HuM1 mice (**Figure 7E**). To confirm the new set of HuM1 and HuM2 mice maintained different gut microbiomes, 16S sequencing of the new fecal samples was performed (**Supplementary Figures 3A–E**). These data suggest a potential systemic increase in the Th17 phenotype in the HuM2 mice due to the composition of the gut microbiota.

### 3.8 Metabolic and cell signaling differences in colonic immune cells of HuM mice

**Figure 8:**
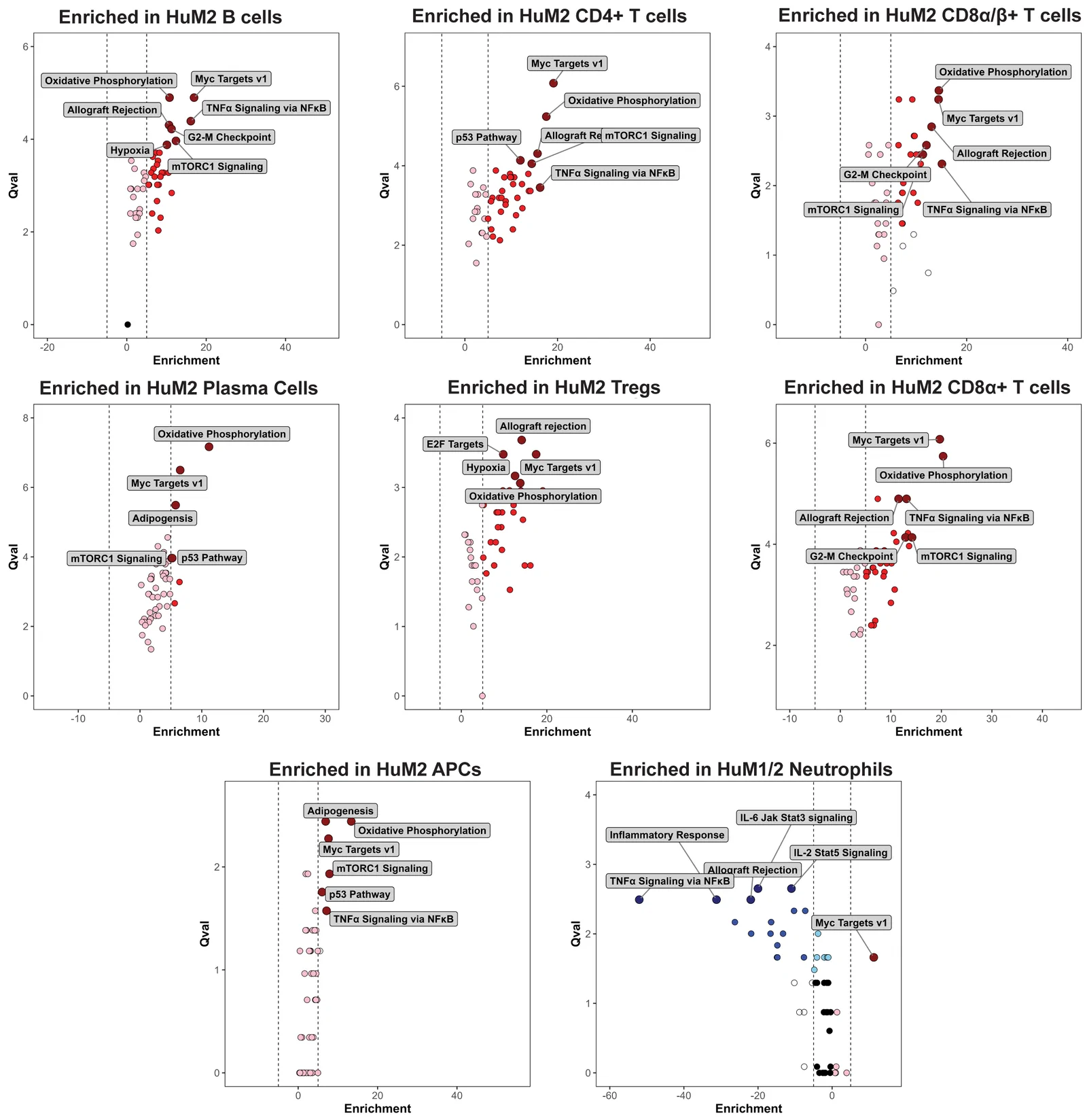
Pathway analysis reveals significant differences in hallmark gene sets between HuM1 and HuM2 samples across multiple cell types. Single Cell Pathway Analysis (SCPA) (v1.6.1) was used to compare pathways from the Molecular Signatures Database (MSigDB) hallmark gene sets between HuM1 and HuM2 samples. Graphs are Qval verses Enrichment [- Fold Change (FC)]. Blue = FC > 5 & adjPval (adjusted p-value) < 0.01, light blue = FC < 5 & FC > 0 & adjPval < 0.01, pink = FC <0 & FC >-5, red = FC < -5 & adjPval < 0.01, and black = FC < 5 & FC > -5 & adjPval > 0.01.

Single-Cell Pathway Analysis was used to observe changes in cell signaling pathways associated with mouse orthologues of hallmark gene sets. In most colonic immune cell subsets, more pathways were enriched in HuM2 mice, with only neutrophils having pathways enriched in HuM1 mice (**Figure 8**). Pathways associated with metabolism and growth often had the highest enrichment scores and q-values including Oxidative Phosphorylation (OXPHOS), Myc Targets v1, mTORC1 Signaling, Hypoxia, C2-M Checkpoint, and E2F targets in HuM2 mice (**Figure 8**). Interestingly, while TNFα signaling via NF-κB and allograft rejection were enriched in HuM2 mice T cells, B cells, and APCs, these pathways were enriched in HuM1 mice neutrophils (**Figure 8**).

When predicted cell-cell signaling was analyzed with CellChat, HuM2 colonic immune cells had both a higher number of inferred interactions and interaction strength (**Supplementary Figure 4A**). Predicted incoming interaction strength was higher in HuM2 CD4^+^ T cells, Plasma cells, B cells, and CD8α^+^ T cells than HuM1 cells of the same subsets, and predicted outgoing interaction strength was higher in HuM2 B cells than HuM1 B cells (**Supplementary Figure 4B**). Fewer predicted signaling pathways were enriched in HuM1 colonic immune cells compared to HuM2, with only collagen, periostin, sell, annexin, vista, PRPRM, and RANKL being enriched in HuM1 (**Supplementary Figure 4C**). HuM2 had an enrichment in 53 cell-cell signaling pathways with notable trends being cytokine signaling (IFN-I, INF-II, TNF, IL16, IL4, MIF, TGFB), cell trafficking (ITGAL-ITGAM, ICAM, SELPLG, CCL), activation/exhaustion (MHCII, PD-L1, PD-L2, FASLG, TRAIL, TIGIT, CD80) (**Supplementary Figure 4C**). Overall, colonic immune cells of HuM2 mice appear to have greater enrichment in metabolic and signaling pathways than those of HuM1 mice.

## 4 Discussion

The microbiota is an important factor in the pathogenesis and therapeutic response to many diseases including cancer. However, as the lab mouse and human gut microbiota have different compositions, using mice with human microbiomes can provide more accurate modeling of microbiota-immune-disease interactions. The purpose of the study was to map the differences in microbiota, metabolites and immune cells in two HuM lines to tease apart microbial-immune interactions that may result in the previously observed differences in response to glioma immunotherapy. HuM1 mice (previous nonresponders) had microbiota dominated by gram-negative taxa such as Bacteroidota, *Alistipes* and *Bacteroides*, and HuM2 mice (previous responders) had microbiota dominated by gram-positive taxa such as Firmicutes, *Ruminococcus, and Bifidobacteria* (**Figure 1**; **Supplementary Figures 1**, **3**). Consistent with previous research, the colons of both lines were dominated by cells of the B cell lineage followed by CD4 T cells (**Figure 4**) (39). However, colons of HuM1 mice had higher frequencies of CD8 T cells and ILCs, whereas HuM2 mice had higher frequencies of B cells and neutrophils (**Figure 5**). Despite the trend for a decrease in frequencies of ILC3s and CD4 T cells in HuM2 colons, these cells had a greater expression of Th17 cytokines. Similarly, HuM2 mice had an increase in Th17 cells in both the gut draining and cervical LNs (**Figure 7**).

Certain gram-positive taxa were enriched in HuM2 fecal samples including *Bifidobacteria* and *Ruminococcus* (**Figure 1**; **Supplementary Figure 3**). Bifidobacterium is a common commensal bacteria known for both pro- and anti-inflammatory immunomodulation (40). Heat killed *Bifidobacterium* promoted DC maturation, T cell proliferation, and IFNγ secretion (41). *B. pseudolongum* has been shown to increase anti-tumor immunity and immunotherapy efficacy through inosine production and exopolysaccharide-TLR2 signaling (42, 43). *Ruminococcus* species can be both inflammatory and anti-cancer. *R. gnavus* is enriched in various inflammatory disorders such as allergies, IBD, and lupus (44–46), while *R. bromii* has been linked to a favorable immunotherapy response for various cancers (47, 48). The descriptive observations from shotgun sequence of a single pooled (n=4) HuM2 fecal sample displayed high abundance of *Turicibacter 1E2* (**Supplementary Figure 1**). Like *Bifidobacteria* and *Ruminococcus*, *Turicibacter* has been linked to favorable outcomes to cancer immunotherapy, but also an increase in immune related adverse events (49).

Alternatively, HuM1 gut microbiota were enriched in *Alistipes*, *Bacteroides* (such as *B. acidifaciens*), (**Figure 1**; **Supplementary Figures 1**, **3**). Like many of the genera discussed*, Alistipes* species have been shown to have both positive and negative effects on immune conditions. *A. finegoldi* has been shown to promote colorectal cancer through the Il-6/Stat3 pathway, while *A. putredinis* was associated with improved immunotherapy response (50, 51). Another study found that *A. shahii* improved CpG-oligodeoxynucleotide therapy for cancers and was correlated with an increase in TLR4 signaling and TNF production (52). *Alistipes* produce LPS (a predicted enriched pathway in HuM1), a major activator of TLR4. *B. acidifaciens* has been shown to increase mucin biosynthesis, and another study found that oral *B. acidifaciens* treatment alleviated DSS-induced colitis and improved gut barrier integrity (53, 54). The descriptive observations from shotgun sequence of a single pooled (n=4) HuM1 fecal sample showed a high abundance of *Dubosiella newyorkensis* (**Supplementary Figure 1**). *D. newyorkensis*, a gram-positive microbe, was also found to alleviate colitis by Treg/Th17 responses and improving mucosal barrier through the production of propionate (55).

Predicted pathway analysis of HuM2 gut microbiota found an enrichment in the pathways that create LTA (glycerolipid metabolism and terpenoid backbone biosynthesis) and peptidoglycan (PG) biosynthesis (**Figure 2**). This is consistent with the observed enrichment in gram-positive bacteria in those mice. LTA and PG both signal through TLR2 and we observed an enrichment in *Tlr2* mRNA in HuM2 colons compared to HuM1 (**Figure 6**). LTA has been shown to activate neutrophils and delay their apoptosis by signaling through TLR2 signaling (56). This may partially account for the slight increase in colonic neutrophils seen in HuM2 mice. TLR2 has been observed on activated CD8 T cells and acting as a costimulatory receptor on these cells (33–35) suggesting the increase in *Tlr2* expressing CD8 T cells in HuM2 colons may be due to differences in activation or stimulation. TLR2 was shown to regulate the differentiation and cytokine profile of B cells (36, 37). LTA administration has been shown to improve anti-tumor immune response in murine UVB-induced skin tumors and a 2024 study found that *TLR2* expression was associated with immune infiltration into GBM tumors (57, 58). Thus, LTA-TLR2 signaling may potentially contribute to the glioma immunotherapy response previously observed in HuM2 mice.

The ceca of HuM1 mice were enriched in acetate and propionate (**Figure 3C**). Propionate has been shown to be associated with Bacteroidota levels (59). This aligns with what we observed with HuM1 mice being enriched in Bacteroidota. Various Bacteroidota can produce propionate, however some Firmicutes members can also produce propionate (60), and propionate has been shown to decrease IL-17 production by γδT cells (61). Furthermore, propionate supplementation reduced Th17, Th1, and neutrophils in the mesenteric LNs, ocular draining LNs, and cornea in mice with herpetic ocular lesions (62). Thus, lower propionate levels may contribute to the systemic Th17 increase observed in HuM2 mice. Butyrate is primarily produced by Lachnospiraceae and Ruminococcaceae members, with some *Odoribacter* species also contributing (63). These taxa of bacteria are in a larger abundance in HuM2 mice, and, while concentration of butyrate in the cecal matter was not different between HuM1 and HuM2 mice, proportionally HuM2 mice had higher butyrate levels (24.92% vs 12.96%) (**Figure 3A**). Butyrate has both anti-inflammatory and immune boosting effects, and it is known to induce Tregs through HDAC inhibition (64). Alternatively, serum butyrate correlated with the efficacy of anti-PD-1 therapy for NSCLC (65), and supplementation enhanced the anti-tumor response in mouse melanoma (65).

Lastly, HuM mice had differences in gut bacteria metabolism and colonic immune cell metabolism. OXPHOS was enriched in HuM2 for many immune cell subsets (**Figure 8**). OXPHOS is often increased in T cells during activation (66), which is in line with the upregulation in activation pathways seen in the HuM2 T cells (**Supplementary Figure 4**). Emerging evidence shows that microbial communities can potentially regulate immune function through metabolic reprogramming, where microbiota-derived metabolites such as butyrate influence glycolysis, fatty acid oxidation, and OXPHOS to shape immune cell activation, differentiation, and effector function (67). For example, butyrate, which had a trend for higher proportions in HuM2, has been shown to increase mitochondrial function and OXPHOS components in various disease models (68, 69). Furthermore, pathway analysis from the microbiota shows a predicted enrichment in HuM2 in glycolysis/gluconeogenesis, pyruvate metabolism, and fatty acid biosynthesis, which would produce metabolites T cells use for OXPHOS (70) (**Figure 2**). OXPHOS is known to be enriched in APCs and plasma cells during colonic inflammation (71). Thus, inflammation could explain the increase in TNFα signaling, and allograft rejection pathways which were enriched in many HuM2 immune cell subsets. Zhou et al. found that OSM signaling through colonic macrophages was associated with inflammation, increased OXPHOS, and resistance to anti-TNF therapy (71). Our CellChat analysis found a predicted enrichment in OSM signaling in HuM2 colonic immune cells (**Supplementary Figure 4**), which may contribute to the OXPHOS, TNFα signaling, and activation we observed. Recent studies have also demonstrated interactions between cellular metabolism, OXPHOS, and the cGAS-STING/type I interferon axis in antitumor immunity, including microbiota-mediated activation of STING signaling (72–74). Although we did not directly assess STING or IFN-I directly, HuM2 mice had an increase in predicted IFN-I signaling in colonic immune cells (**Supplementary Figure 4**). STING signaling activated dendritic cells have been shown to enhance both glycolysis and OXPOHOS (75). Reactive oxygen species generated by OXPHOS can also cause mitochondrial DNA damage that can activate cGAS-STING (76). Thus, interactions of OXPHOS with STING/type I interferon pathways represent a plausible mechanism through which microbiota-driven metabolic reprogramming could influence immune activation and contribute to the previously observed differences in glioma immunotherapy response.

In conclusion, the composition of the microbiota in mice with humanized microbiomes correlates with vastly altered metabolomes and colonic and systemic immune phenotypes. While both lines of HuM mice were genetically identical healthy naïve mice, because the microbiota transplanted were from two different healthy donors, they consequently had significant differences in concentrations of cecal SCFA, and frequencies and phenotypes of colonic B cells, T cells, ILCs, neutrophils, and systemic Th17 cells. Together, the findings highlight a microbiota dependent program associating gram-positive enrichment and altered metabolite production to TLR2-associated immune activation and Th17 response. These differences have the potential to influence the development and treatment of diseases. Although our lab has previously shown that microbial differences in glioma-bearing HuM mice can influence survival and intratumoral immune phenotypes when treated with anti-PD-1 immunotherapy (24, 25), this study uses newly derived HuM offspring mice without corresponding glioma and immunotherapy experiments and therefore cannot establish causality. Recent work using single cell sequencing demonstrated that gut microbiota remodeling with anti-PD-1 therapy reshapes the tumor microenvironment of subcutaneous colon adenocarcinoma models (77). In that study they demonstrated that the gut microbes such as *A. muciniphila* regulated the response to immunotherapy by inhibiting immunosuppressive SPP1^+^ tumor associated macrophages (77). Together, these studies support a broader conceptual framework in which microbiota-dependent baseline immune programming may influence subsequent tumor microenvironment remodeling and immunotherapy responsiveness. Further research with these mice could reveal mechanisms by which the immune response and thus therapy response for various diseases can be altered by human gut microbial composition. These mechanisms may include immune modulation by microbial produced SCFAs, microbial antigen-TLR signaling, or changes in bacterial and host metabolism as observed in the HuM lines discussed. While this study provides important insight into baseline gut microbiota-immune interactions, it is limited by small cohort size, slight microbial shits between mice used for the single cell versus flow cytometry, limited cause-and-effect data, and only baseline measurements. Future experiments will include larger cohort sizes, mechanistic experiments (blocking TLRs, modulating SCFAs, inhibiting specific microbes), and using these HuM mice for modeling the gut microbiota-immune role in disease modulation.

## Data Availability

The datasets presented in this study can be found in online repositories. The names of the repository/repositories and accession number(s) can be found below: https://www.ncbi.nlm.nih.gov/, BioProject ID PRJNA1327931 https://www.ncbi.nlm.nih.gov/, BioProject ID PRJNA1354473.
